# Continuous glycemic monitoring in managing diabetes in adult patients with wolfram syndrome

**DOI:** 10.1007/s00592-024-02350-w

**Published:** 2024-08-03

**Authors:** Agnieszka Zmysłowska, Julia Grzybowska-Adamowicz, Arkadiusz Michalak, Julia Wykrota, Agnieszka Szadkowska, Wojciech Młynarski, Wojciech Fendler

**Affiliations:** 1 Department of Clinical Genetics, Medical University of Lodz, Pomorska Str. 251, Lodz, 92-213 Poland; 2 Department of Pediatrics, Diabetology, Endocrinology and Nephrology, Medical University of Lodz, Lodz, Poland; 3 Department of Biostatistics and Translational Medicine, Medical University of Lodz, Lodz, Poland; 4 Department of Pediatrics, Oncology and Hematology, Medical University of Lodz, Lodz, Poland; 5 Department of Radiation Oncology, Dana-Farber Cancer Institute, Boston, MA USA

**Keywords:** Continuous glucose monitoring system, Glycemic variability, GlyCulator, Type 1 diabetes, Wolfram syndrome

## Abstract

**Aims:**

In this study we evaluated the use of Continuous Glucose Monitoring system in adults with insulin-dependent diabetes in the course of Wolfram syndrome (WFS) in comparison to patients with type 1 diabetes (T1D).

**Methods:**

Individuals with WFS (N = 10) used continuous glucose monitoring for 14 days and were compared with 30 patients with T1D matched using propensity score for age and diabetes duration. Glycemic variability was calculated with Glyculator 3.0.

**Results:**

We revealed significant differences in glycemic indices between adults with Wolfram syndrome-related diabetes and matched comparison group. Patients with Wolfram syndrome presented lower mean glucose in 24-h and nighttime records [24h: 141.1 ± 30.4mg/dl (N = 10) vs 164.9 ± 31.3mg/dl (N = 30), p = 0.0427; nighttime: 136.7 ± 39.6mg/dl vs 166.2 ± 32.1mg/dl (N = 30), p = 0.0442]. Moreover, they showed lower standard deviation of sensor glucose over all periods [24h: 50.3 ± 9.2mg/dl (N = 10) vs 67.7 ± 18.7 mg/dl (N = 30), p = 0.0075; daytime: 50.8 ± 8.7mg/dl (N = 10) vs 67.4 ± 18.0mg/dl (N = 30), p = 0.0082; nighttime: 45.1 ± 14.9mg/dl (N = 10) vs 65.8 ± 23.2mg/dl (n = 30), p = 0.0119] and coefficient of variation at night [33.3 ± 5.8% (N = 10) vs 40.5 ± 8.8% (N = 30), p = 0.0210]. Additionally, WFS patients displayed lower time in high-range hyperglycemia (> 250mg/dl) across all parts of day [24h: 4.6 ± 3.8% (N = 10) vs 13.4 ± 10.5% (N = 30), p = 0.0004; daytime: 4.7 ± 3.9% (N = 10) vs 13.8 ± 11.2% (N = 30), p = 0.0005; nighttime: 4.2 ± 5.5% (N = 10) vs 12.1 ± 10.3% (N = 30), p = 0.0272].

**Conclusions:**

Adult patients with Wolfram syndrome show lower mean blood glucose, less extreme hyperglycemia, and lower glycemic variability in comparison to patients with type 1 diabetes.

**Supplementary Information:**

The online version contains supplementary material available at 10.1007/s00592-024-02350-w.

## Introduction

CGM (Continuous Glucose Monitoring) reduces risk of hypoglycemia and hyperglycemia episodes and enhances the quality of glycemic control [1]. In our previous study, we presented CGM’s utility in children with insulin-dependent diabetes associated with Wolfram syndrome (WFS) [2]. WFS is caused by mutations in the *WFS1* gene and starts with insulin-dependent diabetes, its careful metabolic control prevents diabetes complications and slows the neurodegenerative symptoms progression [3].

The aim of the study was to present a follow-up of the previous work [2] and evaluate CGM-viewed diabetes control in adult patients with WFS compared to patients with type 1 diabetes (T1D).

## Methods

This was a prospective observational substudy on adults with WFS within “TreatWolfram” clinical trial (NCT03717909). The trial was approved by relevant bioethical committee (RNN/379/19/KE) and all participants signed informed consent form at recruitment, including consent for exploratory substudies. For this substudy, available participants were invited to participate during one of outpatient visits. They were provided with the intermittently-scanned CGM FreeStyle Libre 1 (Abbott) system to be assessed during the next follow-up visit. Users were not blinded to the sensors’ readings. Importantly, all participants were treated with insulin at enrollment and were not using CGM on a regular basis prior to enrollment. The diagnosis of WFS was confirmed by molecular tests as previously described [4].

Data obtained from adults with WFS were compared with those from a comparison group including adults with T1D treated in our centre. This group was identified retrospectively, and all generated data originated from standard-of-care procedures, so this part of study was exempt from Bioethical Committee approval. We reviewed our records to identify individuals with T1D that: (1) had type 1 diabetes diagnosed within 2 years of any participant with WFS, (2) used the same brand and model of CGM, (3) had at least 14 days with ≥ 70% of time with available data. Afterwards, we matched those in a ratio of 3:1 to patients with WFS through a propensity score model (PSM) based on age and diabetes duration. These patients constituted the PSM-T1D group (comparison).

For all groups, raw CGM data were downloaded in .csv format. Afterwards, Glyculator 3.0 [5] was used to select 14-day periods with the highest data completeness. Then, we calculated the following glycemic indices according to the definitions in the International Consensus [6]: mean and median sensor glucose (SG), glucose management indicator (GMI), standard deviation (SD), coefficient of variation (CV), time spent in level 2 hypoglycemia below 54 mg/dl (TBR < 54 mg/dl), time spent in at least level 1 hypoglycemia below 70 mg/dl (TBR < 70 mg/dl), time spent in target range between 70 and 180 mg/dl (TIR70-180 mg/dl), time spent in tight range 70-140 mg/dl (TITR70-140 mg/dl), time spent in at least level 1 hyperglycemia above 180 mg/dl (TAR > 180 mg/dl), time spent in level 2 hyperglycemia above 250 mg/dl (TAR > 250 mg/dl), low and high blood glucose indexes (LBGI, HBGI), M100 and J-indexes, mean amplitude of glucose excursion index (MAGE) and glycemic risk assessment diabetes equation score (GRADE).

The analyzed metrics were calculated for the whole-day records (24-h), separately for daytime (6:00–24:00) and nighttime (0:00–6:00). Distribution of the continuous variables across the groups was compared with normal distribution with Shapiro-Wilk tests. Given that most metrics did not significantly deviate from normality, we proceeded with parametric tests and presented data as means and standard deviations. In case of significant deviation from normality, selected analyses were re-done using non-parametric approach (Mann-Whitney U test) as sensitivity analyses. Statistical analyses included between-groups comparison using unpaired t-test for continuous and Fisher’s exact test for nominal data. Comparisons were performed between patients with WFS and matched T1D patients for each GV index and each time-period of data. Due to exploratory nature of the study and multiple collinearities between CGM-based glycemic indices, *p*-values were not corrected for multiple comparisons. In this exploratory setting, the case: comparison ratio was designed to allow for detection (for each variable separately) of between-group differences of magnitude of one standard deviation or larger with 75% power – estimated based on results observed in previous studies in children [2]. The alpha level of 0.05 was considered significant for each comparison. All glycemic indexes were treated with the same priority and, due to exploratory nature of the study, no correction for multiple comparisons was applied. We also performed comparisons between WFS and the whole available group with T1D as a sensitivity analysis.

## Results

We recruited *N* = 10 adults with WFS [mean age 24.2 ± 3.8y.o., mean diabetes duration 17.1 ± 4.8 years]. Their mean HbA1c was 57.6 ± 4.8mmol/mol (7.4 ± 0.4%) (*N* = 9, one measurement missing from an individual with GMI 6.2%) and BMI 25 ± 5.6 kg/m^2 (*N* = 9, one measurement missing). In parallel, a retrospective review of our center’s records identified *N* = 57 adults with T1D as a eligible comparison group – their characteristics is included in Supplementary Table 1. Logistic-regression-based propensity score matching by diabetes duration and age produced a comparison group of *N* = 30 adults with T1D (Supplementary Fig. 1). Not controlled clinical characteristics were comparable between the groups (Table 1). All records from individuals with WFS were collected in 2021, in PSM-T1D group records from 2021 were also a majority (22/30, 73.3%).

**Table 1:** Group characteristics and comparisons

Variable	Time range	WFS *N* = 10	PSM-T1D ^1^ *N* = 30	*p*-value WFS vs. PSM-T1D
Categorical characteristics [N(%)]				
Time of data collection	2020 2021 2022 2023	0 (0%) 10 (100%) 0 (0%) 0 (0%)	3 (10%) 22 (73.3%) 2 (6.7%) 3 (10%)	0.1655
Insulin therapy tools - CSII	-	10 (100%)	24 (80%)	0.3075
CGM used - isCGM	-	10 (100%)	30 (100%)	NA
Continuous characteristics (Mean ± SD)				
Age [years]	-	23.9 ± 3.7	21.4 ± 4.1	0.0989
Diabetes duration [years]	-	17.1 ± 4.8	14.6 ± 4.3	0.1306
HbA1c [%]	-	7.5 ± 0.48	7.6 ± 1.4	0.7838
Daily insulin dose [UI/kg]	-	0.72 ± 0.07	0.78 ± 0.19	0.1644
BMI [kg/m^2]	-	24.5 ± 5.6	22.5 ± 3.5	0.3971
CGM record completeness [% from 14 consecutive days]	-	86.3 ± 11.8	94.4 ± 6.3	0.0635
GMI	24-hours	6.7 ± 0.7	7.3 ± 0.7	**0.0427**
	daytime (6:00–24:00)	6.7 ± 0.7	7.3 ± 0.8	**0.0442**
	nightime (0:00–6:00)	6.6 ± 0.9	7.2 ± 0.8	0.0637
Mean SG [mg/dl]	24-hours	141.1 ± 30.4	164.9 ± 31.3	**0.0427**
	daytime (6:00–24:00)	142.5 ± 28.3	166.2 ± 32.1	**0.0442**
	nightime (0:00–6:00)	136.7 ± 39.6	160.7 ± 32.6	0.0637
CV of SG [%]	24-hours	36.4 ± 6.9	40.9 ± 6.2	0.0597
	daytime (6:00–24:00)	36.3 ± 6.7	40.4 ± 5.7	0.0704
	nightime (0:00–6:00)	33.3 ± 5.8	40.5 ± 8.8	**0.0210**
TBR < 54 mg/dl [%]	24-hours	2.2 ± 2.1	1.3 ± 2.0	0.2644
	daytime (6:00–24:00)	2.1 ± 2.4	1.0 ± 1.5	0.2017
	nightime (0:00–6:00)	2.4 ± 2.8	2.5 ± 4.1	0.9747
TBR < 70 mg/dl[%]	24-hours	8.2 ± 7.3	5.5 ± 4.3	0.2919
	daytime (6:00–24:00)	8.3 ± 8.1	4.8 ± 3.9	0.2194
	nightime (0:00–6:00)	8.0 ± 9.5	7.5 ± 6.8	0.8630
TIR70-140 mg/dl [%]	24-hours	45.4 ± 19.3	38.2 ± 15.1	0.2296
	daytime (6:00–24:00)	44.3 ± 18.2	38.1 ± 15.0	0.2856
	nightime (0:00–6:00)	48.7 ± 23.9	38.5 ± 17.2	0.1516
TIR70-180 mg/dl [%]	24-hours	66.8 ± 13.0	58.3 ± 13.8	0.0933
	daytime (6:00–24:00)	66.3 ± 12.5	58.4 ± 13.9	0.1199
	nightime (0:00–6:00)	68.3 ± 17.0	57.9 ± 15.8	0.0849
TAR > 180 mg/dl [%]	24-hours	25.0 ± 17.2	36.2 ± 15.7	0.0627
	daytime (6:00–24:00)	25.4 ± 16.3	36.7 ± 15.8	0.0581
	nightime (0:00–6:00)	23.7 ± 21	34.6 ± 18.1	0.1225
TAR > 250 mg/dl [%]	24-hours	4.6 ± 3.8	13.4 ± 10.5	**0.0004**
	daytime (6:00–24:00)	4.7 ± 3.9	13.8 ± 11.2	**0.0005**
	nightime (0:00–6:00)	4.2 ± 5.5	12.1 ± 10.3	**0.0272**
MAGE	24-hours	95.0 ± 17.0	130.8 ± 36.5	**0.0051**
	daytime (6:00–24:00)	96.0 ± 16.6	132.8 ± 36.9	**0.0001**
	nightime (0:00–6:00)	85.4 ± 30.8	129.7 ± 49.9	**0.0123**

Abbreviations used: CSII – continuous subcutaneous insulin infusion (personal insulin pump), isCGM – intermittently-scanned continuous glucose monitoring, WFS – Wolfram Syndrome, T1D – type 1 diabetes, PSM-T1D – subset of T1D comparison group matched with propensity-score-matching, BMI – body mass index, GMI – glucose management indicator, SG – sensor glucose, CV – coefficient of variation, TBR - time spent below range, TIR - time spent in target range, TITR – time spent in tight range, TAR - time spent above range, MAGE - mean amplitude of glucose excursion index

1 – matched: first by same CGM technology, same season of CGM data (by hand), then by propensity-score matching for age and diabetes duration (3:1)

*p*-values generated with fisher`s exact test for nominal variables and t-test for continuous characteristics, with equal or unequal variance assumption depending on results of Levene`s test

**Fig. 1:**
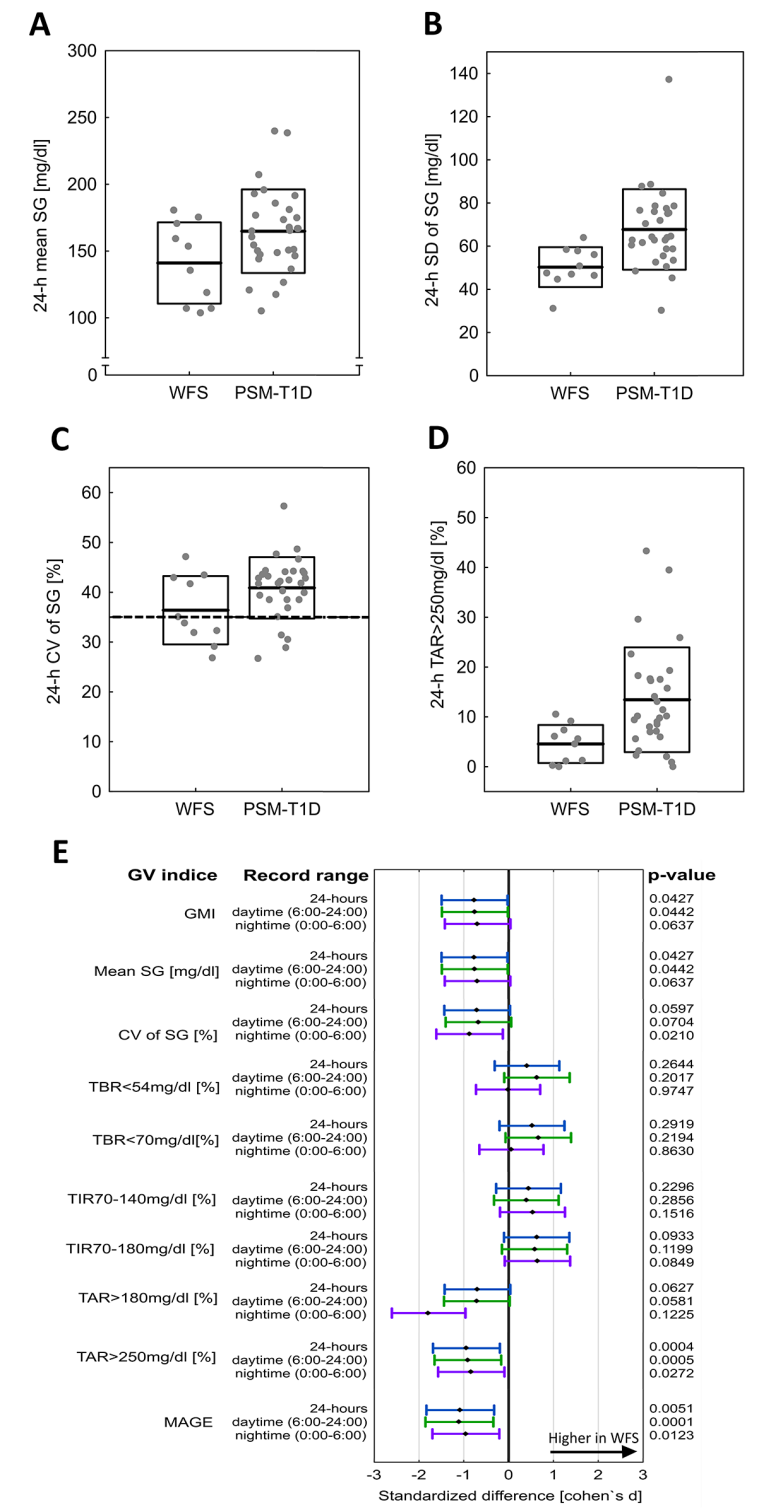
(**A-D**) Box-plots comparing differences in chosen glycemic indices [A: mean sensor glucose (SG), B: standard deviation (SD) of SG, C: coefficient of variation (CV), D: time spent above 250 mg/dl (TAR > 250 mg/dl)] between adults with Wolfram syndrome (WFS, *N* = 10) and propensity-score-matched comparison group with type 1 diabetes (matching for age and diabetes duration at visit, PSM-T1D, *N* = 30). Lines denote means, boxes span one standard deviation. Raw data are depicted with gray dots. For CV, a horizontal dashed line denotes 35%, a guidelines-based value for high glycemic variability. (**E**) Standardized differences in glycemic indices between adults with Wolfram syndrome and type 1 diabetes. Standardized differences were calculated as Cohen’s d with 95% confidence intervals based on non-central t distribution. Black diamonds denote standardized differences, whiskers span 95% confidence intervals, with color corresponding to time ranges used for analysis (blue – 24 h whole day records, green – daytime records 6:00–24:00, purple – nighttime records 0:00–6:00). All comparisons were made on 14-day records from *N* = 10 adults with Wolfram syndrome and *N* = 30 propensity-score-matched adults with type 1 diabetes. Abbreviations used: WFS – Wolfram Syndrome, T1D – type 1 diabetes, PSM-T1D – T1D comparison group matched with propensity-score-matching, GMI – glucose management indicator, SG – sensor glucose, CV – coefficient of variation, TBR - time spent below range, TIR - time spent in target range, TITR – time spent in tight range, TAR - time spent above range, MAGE - mean amplitude of glucose excursion index

Comparison of 14-day CGM records between individuals with WFS and T1D-PSM group revealed marked differences in glycemic indices (see Fig. 1A-D for raw data on select glycemic indices, Fig. 1E for standardized differences and Table 1 for numerical results). Overall, adults with WFS presented lower mean glucose in 24-h and nighttime records [24 h: WFS 141.1 ± 30.4 mg/dl (*N* = 10) vs. T1D-PSM 164.9 ± 31.3 mg/dl (*N* = 30), *p* = 0.0427; nighttime: WFS 136.7 ± 39.6 mg/dl (*N* = 10) vs. T1D-PSM 166.2 ± 32.1 mg/dl (*N* = 30), *p* = 0.0442]. The most notable difference was that WFS group displayed lower time in level 2 hyperglycemia TAR > 250 mg/dl, which was consistent over all periods [24 h: WFS 4.6 ± 3.8% (*N* = 10) vs. T1D-PSM 13.4 ± 10.5% (*N* = 30), *p* = 0.0004; daytime: WFS 4.7 ± 3.9% (*N* = 10) vs. T1D-PSM 13.8 ± 11.2% (*N* = 30), *p* = 0.0005; nighttime: WFS 4.2 ± 5.5% (*N* = 10) vs. T1D-PSM 12.1 ± 10.3% (*N* = 30), *p* = 0.0272]. This differences corresponded with overall hyperglycemia index HBGI [24-h: WFS 5.1 ± 3.3 (*N* = 10) vs. T1D-PSM 8.9 ± 5.2 (*N* = 30), *p* = 0.0367, and to a lesser extent with time in at least level 1 hyperglycemia TAR > 180 mg/dl [24 h: WFS 25.0 ± 17.2% (*N* = 10) vs. T1D-PSM 36.2 ± 15.7% (*N* = 30), *p* = 0.0627].

In terms of glycemic variability, WFS group presented lower SD of sensor glucose (SG) across all assessed time periods. However, CV which is independent of mean glucose, was lower only during nighttime. [24 H: SD - WFS 50.3 ± 9.2 mg/dl (*N* = 10) vs. T1D-PSM 67.7 ± 18.7 mg/dl (*N* = 30), *p* = 0.0075; CV − 36.4 ± 6.9% vs. 40.9 ± 6.2%, *p* = 0.0597; daytime: SD - WFS 50.8 ± 8.7 mg/dl (*N* = 10) vs. T1D-PSM 67.4 ± 18.0 mg/dl (*N* = 30), *p* = 0.0082; CV − 36.3 ± 6.7% vs. 40.4 ± 5.7%, *p* = 0.0704; nighttime: SD - WFS 45.1 ± 14.9 mg/dl (*N* = 10) vs. T1D-PSM 65.8 ± 23.2 mg/dl (*N* = 30), *p* = 0.0119; CV − 33.3 ± 5.8% (*N* = 10) vs. T1D-PSM 40.5 ± 8.8% (*N* = 30), *p* = 0.0210]. Most of detected differences were also present when contrasting adults with WFS with pre-matching T1D group (Supplementary Table 1), and sensitivity analyses with non-parametric tests produced consistent results [data not shown].

## Discussion

Adults with WFS tend to experience fewer level 2 hyperglycemic episodes than those with T1D, with no difference in the rate of hypoglycemia < 70 mg/dl, which was present in children with WFS [2]. In line with that, longer time in hyperglycemia > 250 mg/dl was reported in patients with T1D. We noted that in five individuals in this group 20% of all measurements were > 250 mg/dl. Such occurrences were absent in the WFS group. These findings correspond with previously reported lower daily insulin requirements and lower HbA1c levels in patients with WFS than in patients with type 1 diabetes [7] and with our previous study in children [2].

A limitation of the study is relatively small number of participants studied, since WFS is a very rare disease [3]. Thus, due to the number of compared variables and lack of significance threshold correction, it is possible that false-positive associations were present more often than in 5% of tests, and the results should be treated as exploratory. However, direction of differences and consistency between similar metrics diminishes this risk. Secondly, the observation period was quite short, but it was dictated by the capacity of patients to maintain proper sensor use despite their disabilities associated with WFS (loss of visual acuity). Longer observation would be useful, but the selection of the optimal CGM system for patients with WFS remains debatable. In conclusion, our study provided insight into glycemic outcomes in adult patients with WFS and showed differences in some glycemic indexes compared to those with T1D. The non-autoimmune course of diabetes in WFS appears to be associated with lower and more stable glucose levels. Over time, both groups may develop complications of diabetes. The older the patients with WFS, the more diabetes-related complications can overlap with progressive neurodegeneration, which remains a key problem in this syndrome and a cause of their premature death [3]. Thus, continuous glycemic monitoring remains a useful tool to monitor glycemic control in this group with great detail. Due to observational nature of our study, we cannot directly link CGM use or more aggressive insulin treatment it permits with the progress of neurodegeneration. However, benefits of CGM should be considered when when choosing precise insulin dosing in adults with WFS.

## Electronic supplementary material

Below is the link to the electronic supplementary material.

Supplementary Figure 1: Flowchart presenting recruitment of the studied group of adults with Wolfram syndrome (WFS) and identification and subsequent matching of comparison group including adults with type 1 diabetes (T1D)

Supplementary Table 1: Group characteristics and comparisons

## Data Availability

The data may be available to all interested persons, upon request to the correspondent author.
