# Supplementary figures and images for: Continuous glycemic monitoring in managing diabetes in adult patients with wolfram syndrome
Source: Acta Diabetol. 2024 Aug 3;61(10):1333–8. doi: 10.1007/s00592-024-02350-w (PMC11486770; doi:10.1007/s00592-024-02350-w)

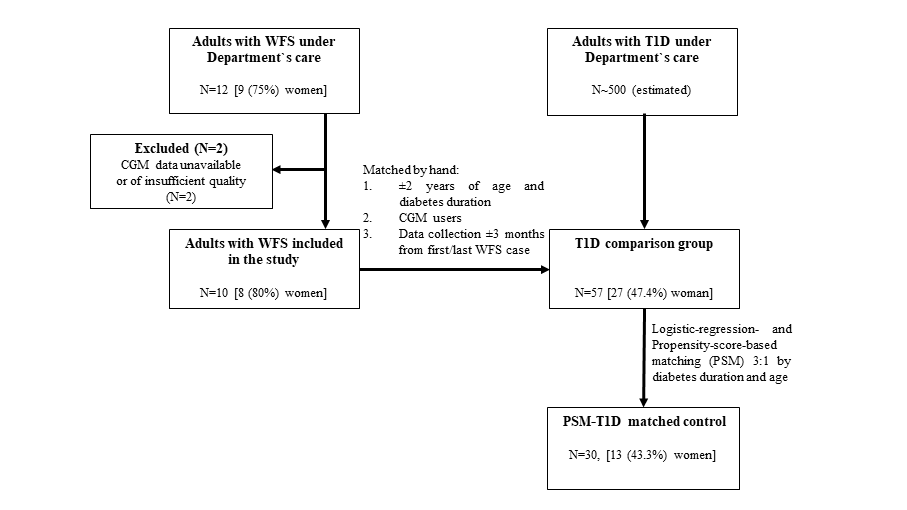

Supplement: Supplementary file 1 — Supplementary Figure 1: Flowchart presenting recruitment of the studied group of adults with Wolfram syndrome (WFS) and identification and subsequent matching of comparison group including adults with type 1 diabetes (T1D) [file 592_2024_2350_MOESM1_ESM.png]
