# Supplementary material for: Continuous glycemic monitoring in managing diabetes in adult patients with wolfram syndrome
Source: Acta Diabetol. 2024 Aug 3;61(10):1333–8. doi: 10.1007/s00592-024-02350-w (PMC11486770; doi:10.1007/s00592-024-02350-w)
Supplement: Supplementary file 2 — Supplementary Table 1: Group characteristics and comparisons [file 592_2024_2350_MOESM2_ESM.docx]

**Supplementary Table 1. Group characteristics and comparisons**

| Variable | Time range | WFS  N=10 | T1D patients 1  N=57 | p-value WFS vs T1D | PSM-T1D comparison group 2  N=30 | p-value  WFS vs PSM-T1D |
| --- | --- | --- | --- | --- | --- | --- |
| Categorical characteristics [N(%)] | | | | | | |
| **Time of data collection** | **2020**  **2021**  **2022**  **2023** | 0 (0%)  10 (100%)  0 (0%)  0 (0%) | 4 (7\|%)  48 (84.2%)  2 (3.5%)  3 (5.3%) | 0.3353 | 3 (10%)22 (73.3%)  2 (6.7%)  3 (10%) | 0.1655 |
| **Insulin therapy tools - CSII** | - | 10 (100%) | 46 (80.7%) | 0.1946 | 24 (80%) | 0.3075 |
| **CGM used- isCGM** | - | 10 (100%) | 57 (100%) | NA | 30 (100%) | NA |
| Continuous characteristics (Mean±SD) | | | | | | |
| **Age [years]** | - | 23.9±3.7 | 18.2±4.6 | **0.0004** | 21.4±4.1 | 0.0989 |
| **Diabetes duration [years]** | - | 17.1±4.8 | 11.9±4.3 | **0.0010** | 14.6±4.3 | 0.1306 |
| **HbA1c [%]** | - | 7.5±0.48 | 7.5±1.0 | 0.8923 | 7.6±1.4 | 0.7838 |
| **Daily insulin dose [UI/kg]** | - | 0.72±0.07 | 0.87±0.28 | **0.0112** | 0.78±0.19 | 0.1644 |
| **BMI [kg/m^2]** | - | 24.5±5.6 | 22.5±3.5 | 0.3073 | 22.5±3.5 | 0.3971 |
| **CGM record completeness**  **[% from 14 consecutive days]** |  | 86.3±11.8 | 94.4±5.9 | 0.0609 | 94.4±6.3 | 0.0635 |
| **GMI** | 24-hours | 6.7±0.7 | 7.3±0.9 | **0.0305** | 7.3±0.7 | **0.0427** |
|  | daytime (6:00-24:00) | 6.7±0.7 | 7.4±0.9 | **0.0246** | 7.3±0.8 | **0.0442** |
|  | nightime (0:00-6:00) | 6.6±0.9 | 7.0±0.9 | 0.1273 | 7.2±0.8 | 0.0637 |
| **Mean SG [mg/dl]** | 24-hours | 141.1±30.4 | 167.6±35.7 | **0.0305** | 164.9±31.3 | **0.0427** |
|  | daytime (6:00-24:00) | 142.5±28.3 | 171.3±37.7 | **0.0246** | 166.2±32.1 | **0.0442** |
|  | nightime (0:00-6:00) | 136.7±39.6 | 156.0±36.0 | 0.1273 | 160.7±32.6 | 0.0637 |
| **Median SG [mg/dl]** | 24-hours | 133.7±35.1 | 158.0±35.7 | 0.0506 | 154.5±30.6 | 0.0805 |
|  | daytime (6:00-24:00) | 135.5±32.2 | 162.3±38.4 | **0.0417** | 155.9±31.8 | 0.0883 |
|  | nightime (0:00-6:00) | 130.8±41.1 | 148.3±36.5 | 0.1744 | 151.9±30.5 | 0.0904 |
| **SD of SG [mg/dl]** | 24-hours | 50.3±9.2 | 68.9±20.3 | **0.0062** | 67.7±18.7 | **0.0075** |
|  | daytime (6:00-24:00) | 50.8±8.7 | 69.0±20.3 | **<0.0001** | 67.4±18.0 | **0.0082** |
|  | nightime (0:00-6:00) | 45.1±14.9 | 62.2±23.3 | **0.0289** | 65.8±23.2 | **0.0119** |
| **CV of SG [%]** | 24-hours | 36.4±6.9 | 40.9±6.7 | 0.0546 | 40.9±6.2 | 0.0597 |
|  | daytime (6:00-24:00) | 36.3±6.7 | 40.1±6.1 | 0.0770 | 40.4±5.7 | 0.0704 |
|  | nightime (0:00-6:00) | 33.3±5.8 | 39.6±10.8 | 0.0757 | 40.5±8.8 | **0.0210** |
| **TBR<54mg/dl [%]** | 24-hours | 2.2±2.1 | 1.6±2.4 | 0.5001 | 1.3±2.0 | 0.2644 |
|  | daytime (6:00-24:00) | 2.1±2.4 | 1.1±1.5 | 0.2366 | 1.0±1.5 | 0.2017 |
|  | nightime (0:00-6:00) | 2.4±2.8 | 3.3±5.5 | 0.6260 | 2.5±4.1 | 0.9747 |
| **TBR<70mg/dl[%]** | 24-hours | 8.2±7.3 | 5.9±4.9 | 0.1974 | 5.5±4.3 | 0.2919 |
|  | daytime (6:00-24:00) | 8.3±8.1 | 4.9±4.1 | 0.2224 | 4.8±3.9 | 0.2194 |
|  | nightime (0:00-6:00) | 8.0±9.5 | 8.8±8.7 | 0.7896 | 7.5±6.8 | 0.8630 |
| **TIR70-140mg/dl [%]** | 24-hours | 45.4±19.3 | 37.2±15.2 | 0.1316 | 38.2±15.1 | 0.2296 |
|  | daytime (6:00-24:00) | 44.3±18.2 | 36.3±15.1 | 0.1371 | 38.1±15.0 | 0.2856 |
|  | nightime (0:00-6:00) | 48.7±23.9 | 39.8±18.5 | 0.1866 | 38.5±17.2 | 0.1516 |
| **TIR70-180mg/dl [%]** | 24-hours | 66.8±13.0 | 57.0±15.7 | 0.0656 | 58.3±13.8 | 0.0933 |
|  | daytime (6:00-24:00) | 66.3±12.5 | 56.3±16.0 | 0.0668 | 58.4±13.9 | 0.1199 |
|  | nightime (0:00-6:00) | 68.3±17.0 | 59.2±18.4 | 0.1489 | 57.9±15.8 | 0.0849 |
| **TAR>180mg/dl [%]** | 24-hours | 25.0±17.2 | 37.1±17.6 | **0.0466** | 36.2±15.7 | 0.0627 |
|  | daytime (6:00-24:00) | 25.4±16.3 | 38.8±18.1 | **0.0320** | 36.7±15.8 | 0.0581 |
|  | nightime (0:00-6:00) | 23.7±21 | 32±20.3 | 0.2394 | 34.6±18.1 | 0.1225 |
| **TAR>250mg/dl [%]** | 24-hours | 4.6±3.8 | 14.7±12.8 | **<0.0001** | 13.4±10.5 | **0.0004** |
|  | daytime (6:00-24:00) | 4.7±3.9 | 15.8±13.9 | **<0.0001** | 13.8±11.2 | **0.0005** |
|  | nightime (0:00-6:00) | 4.2±5.5 | 11.0±12.0 | 0.0853 | 12.1±10.3 | **0.0272** |
| **LBGI** | 24-hours | 2.0±1.6 | 1.4±1.1 | 0.1565 | 1.3±1.0 | 0.2403 |
|  | daytime (6:00-24:00) | 1.9±1.6 | 1.2±0.9 | 0.2012 | 1.2±0.9 | 0.2061 |
|  | nightime (0:00-6:00) | 2.3±2.3 | 2.1±2.2 | 0.7913 | 1.8±1.7 | 0.4424 |
| **HBGI** | 24-hours | 5.1±3.3 | 9.4±6.1 | **0.0333** | 8.9±5.2 | **0.0367** |
|  | daytime (6:00-24:00) | 5.1±3.1 | 10.0±6.6 | **0.0276** | 9.0±5.3 | **0.0365** |
|  | nightime (0:00-6:00) | 4.9±4.1 | 7.7±5.7 | 0.1398 | 8.4±5.2 | 0.0630 |
| **M100** | 24-hours | 181.1±49.7 | 224.6±65.2 | **0.0494** | 218.5±57.7 | 0.0750 |
|  | daytime (6:00-24:00) | 182.2±46.7 | 229.1±68.3 | **0.0413** | 219.3±58.8 | 0.0786 |
|  | nightime (0:00-6:00) | 177.8±61.7 | 210.3±67.4 | 0.1598 | 216.0±59.8 | 0.0905 |
| **J-index** | 24-hours | 37.8±13.8 | 58.7±27.9 | **0.0244** | 56.3±24.2 | **0.0284** |
|  | daytime (6:00-24:00) | 38.4±12.9 | 60.8±29.8 | **0.0231** | 56.8±24.6 | **0.0302** |
|  | nightime (0:00-6:00) | 35.5±18.6 | 50.5±25.7 | 0.0823 | 53.9±25.2 | **0.0402** |
| **MAGE** | 24-hours | 95.0±17.0 | 131.7±39.0 | **<0.0001** | 130.8±36.5 | **0.0051** |
|  | daytime (6:00-24:00) | 96.0±16.6 | 133.8±40.4 | **<0.0001** | 132.8±36.9 | **0.0001** |
|  | nightime (0:00-6:00) | 85.4±30.8 | 121.0±48.4 | **0.0283** | 129.7±49.9 | **0.0123** |
| **Grade** | 24-hours | 7.3±2.9 | 10.1±4.0 | **0.0386** | 9.7±3.5 | 0.0590 |
|  | daytime (6:00-24:00) | 7.4±2.7 | 10.3±4.3 | **0.0402** | 9.7±3.6 | 0.0719 |
|  | nightime (0:00-6:00) | 7.0±3.8 | 9.4±4.1 | 0.0792 | 9.7±3.6 | **0.0481** |

1 – matched by hand: ±2 years of age and diabetes duration, same CGM technology, same season of CGM data

2 – matched: first by same CGM technology, same season of CGM data (by hand), then by propensity-score matching for age and diabetes duration (3:1)

p-values generated with fisher`s exact test for nominal variables and t-test for continuous characteristics, with equal or unequal variance assumption depending on results of Levene`s test.

Abbreviations used: CSII – continuous subcutaneous insulin infusion (personal insulin pump), isCGM – intermittently-scanned continuous glucose monitoring, WFS – Wolfram Syndrome, T1D – type 1 diabetes, PSM-T1D – T1D comparison group matched with propensity-score-matching, GMI – glucose management indicator, SG – sensor glucose, SD – standard deviation, CV – coefficient of variation, TBR - time spent below range, TIR - time spent in target range, TITR – time spent in tight range, TAR - time spent above range, LBGI - low blood glucose index, HBGI - high blood glucose index, MAGE - mean amplitude of glucose excursion index, GRADE - glycemic risk assessment diabetes equation score.
